# Supplementary material for: Restriction of Glycolysis Increases Serial Killing Capacity of Natural Killer Cells
Source: Int J Mol Sci. 2024 Mar 2;25(5):2917. doi: 10.3390/ijms25052917 (PMC10932276; doi:10.3390/ijms25052917)
Supplement: Supplementary file 1 [file ijms-25-02917-s001.zip › ijms-2885306-supplementary.pdf]

International Journal of *Molecular Sciences*

MDPI

Supplementary Materials

Restriction of Glycolysis Increases Serial Killing Capacity of Natural Killer Cells

Lea Katharina Picard [1], Jens Alexander Niemann [1], Elisabeth Littwitz-Salomon [2], Herbert Waldmann [3,4] and Carsten Watzl [1,*]

[1] Department for Immunology, Leibniz Research Centre for Working Environment and Human Factors at TU Dortmund (IfADo), D-44139 Dortmund, Germany; lea.picard@uk-koeln.de (L.K.P.); niemann@ifado.de (J.A.N.)
[2] Institute for Virology, Institute for Translational HIV Research, University Hospital Essen, D-45147 Essen, Germany; elisabeth.littwitz@uni-due.de
[3] Max Planck Institute of Molecular Physiology, D-44227 Dortmund, Germany; herbert.waldmann@mpi-dortmund.mpg.de
[4] Faculty of Chemistry, Chemical Biology, Technical University Dortmund, D-44227 Dortmund, Germany
* Correspondence: watzl@ifado.de

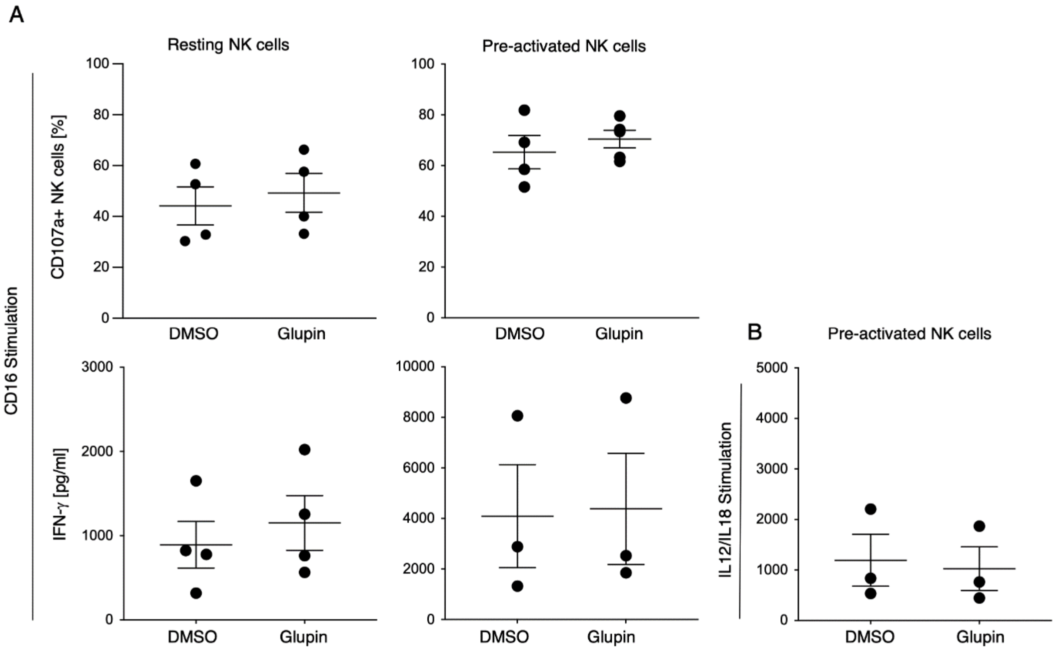

Figure S1. NK cells can perform their functions despite short-term inhibition of glucose uptake. (A) Resting or preactivated NK cells were stimulated after pretreatment with 100 nM Glupin or DMSO for 30 min via plate-bound CD16 for 2 h (CD107a assay) or 16 h (IFN- ELISA). n=3-4. Data were pooled from three or four independent experiments, each experiment using one donor. (B) Preactivated NK cells were stimulated with IL-12 and IL-18 for 16 h after pretreatment with 100 nM Glupin or DMSO for 30 min. IFN-g was determined by ELISA. n=3. Data were pooled from three independent experiments, with each experiment performed with one donor. Mean with SEM.

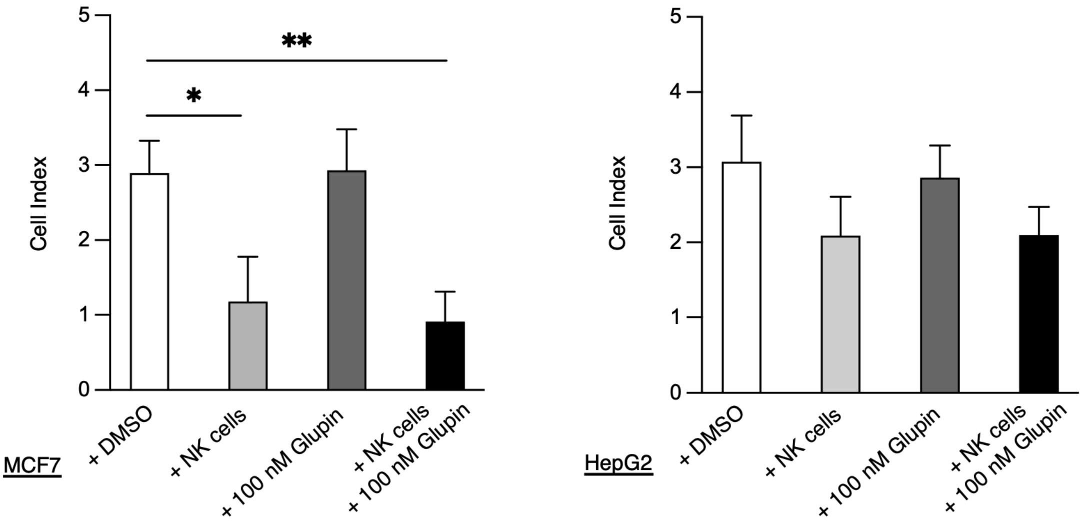

Figure S2. No negative effect of Glupin on NK cell cytotoxicity. RCTA analysis of MCF7 cells (left) and HepG2 cells (right) in the presence or absence of NK cells, 100 nM Glupin or NK cells + 100 nM Glupin. Cell index data shown were obtained 72 hours after addition of NK cells +/- Glupin. n=3. Data were pooled from three independent experiments, using one donor for each experiment. Mean with SEM. Statistics: paired t-test. Significant differences are indicated by asterisks *P ≤ 0.05, **P ≤ 0.01.

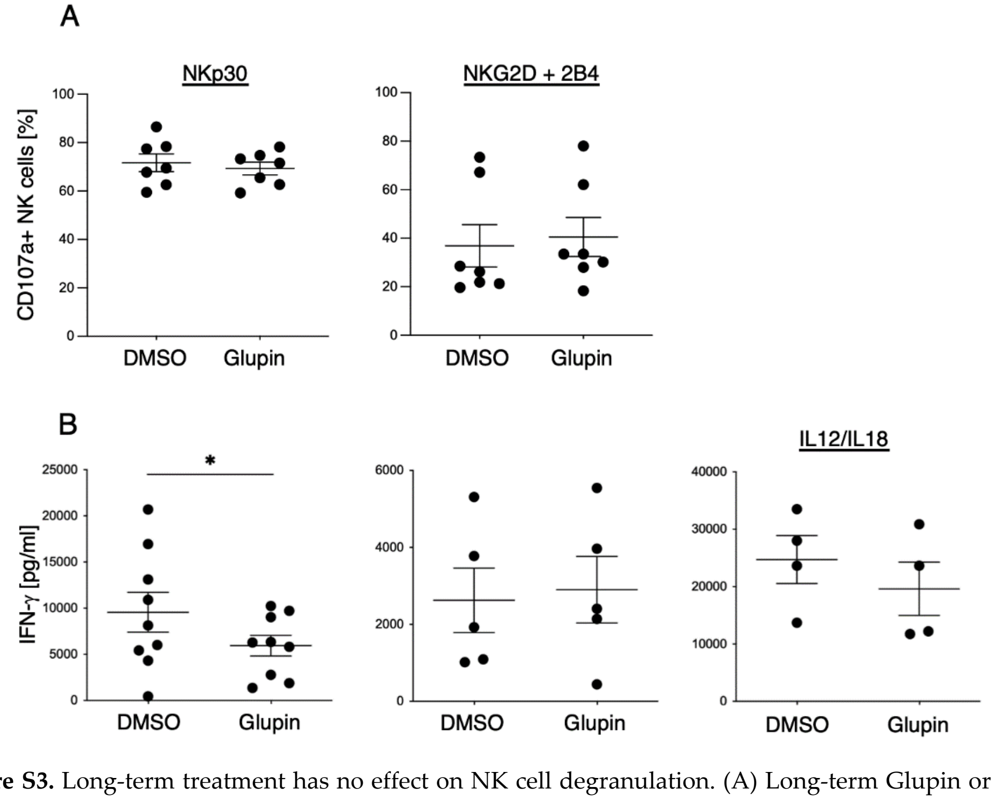

Figure S3. Long-term treatment has no effect on NK cell degranulation. (A) Long-term Glupin or DMSO treated NK cells were stimulated with plate-bound antibodies against NKp30 or NKG2D + 2B4 for 2 hours and then assessed for degranulation. n=7. Data were pooled from seven independent experiments, each experiment using one donor. (B) Long-treated NK cells were stimulated with plate-bound antibodies against NKp30, or NKG2D + 2B4 or with IL-12 + IL-18 for 16 hours, and IFN-γ secretion was measured by ELISA. n=4-9. Data were pooled from four to nine independent experiments, with each experiment performed with one donor. Mean with SEM. Statistics: paired t test. Significant differences are indicated by asterisks *P ≤ 0.05.

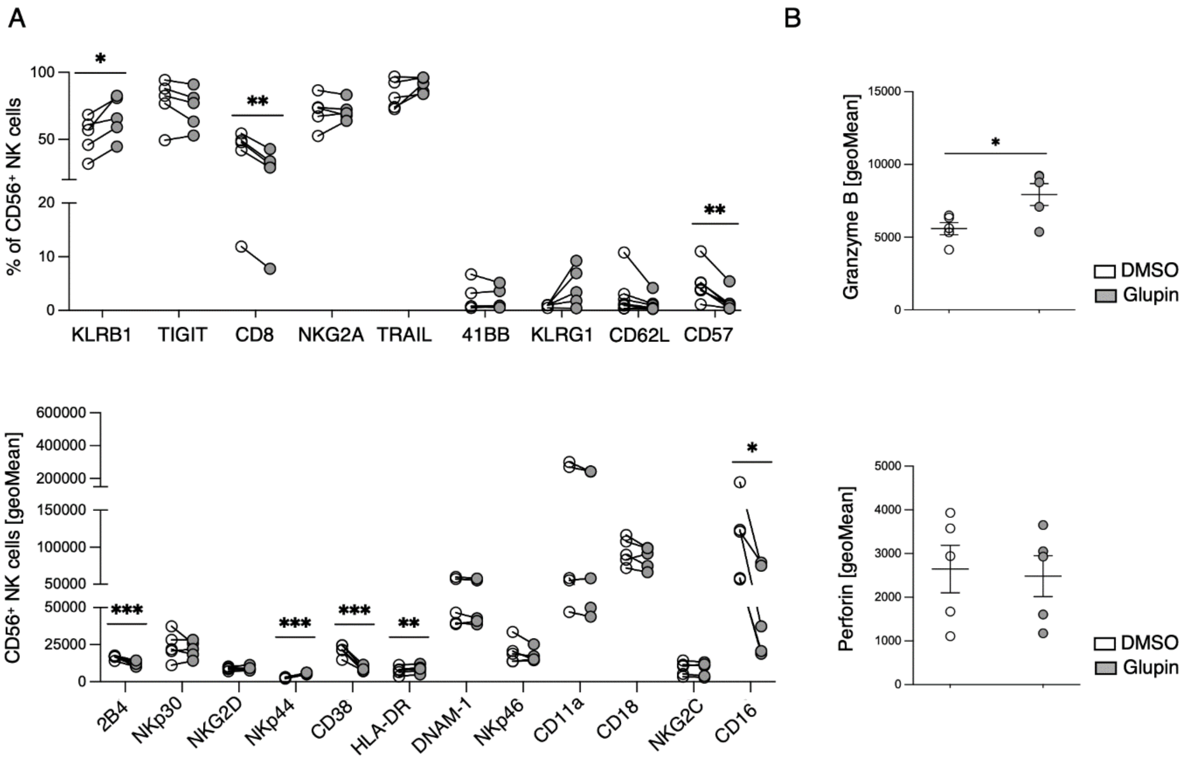

Figure S4. Long-term treatment with Glupin results in altered phenotype of NK cells. (A) The expression level of the indicated surface molecules was analyzed on day 21 of long-term Glupin or DMSO treated NK cells. n=5. Data were pooled from five independent experiments, each experiment using one donor. Statistics: paired t-test. (B) Granzyme B and perforin expression in long-term treated NK cells was analyzed by flow cytometry. n=5. Data were pooled from five independent experiments, with each experiment performed with one donor. Mean with SEM. Statistics: paired t-test. Significant differences are indicated by asterisks *P ≤ 0.05, **P ≤ 0.01, ***P ≤ 0.001.

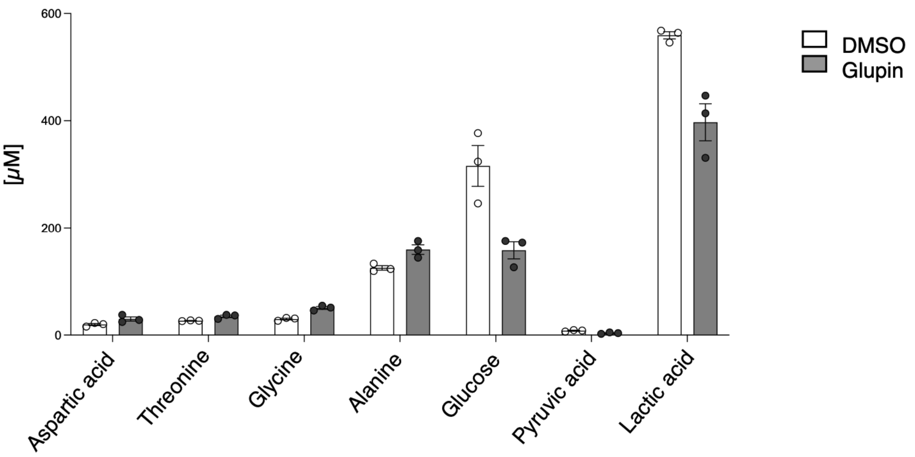

Figure S5. Metabolomics of long-term treated NK cells. Whole lysate of long-term Glupin or DMSO treated NK cells were used for metabolomics. n=3. Data were pooled from three independent experiments each experiment was performed with one donor. Mean with SEM.

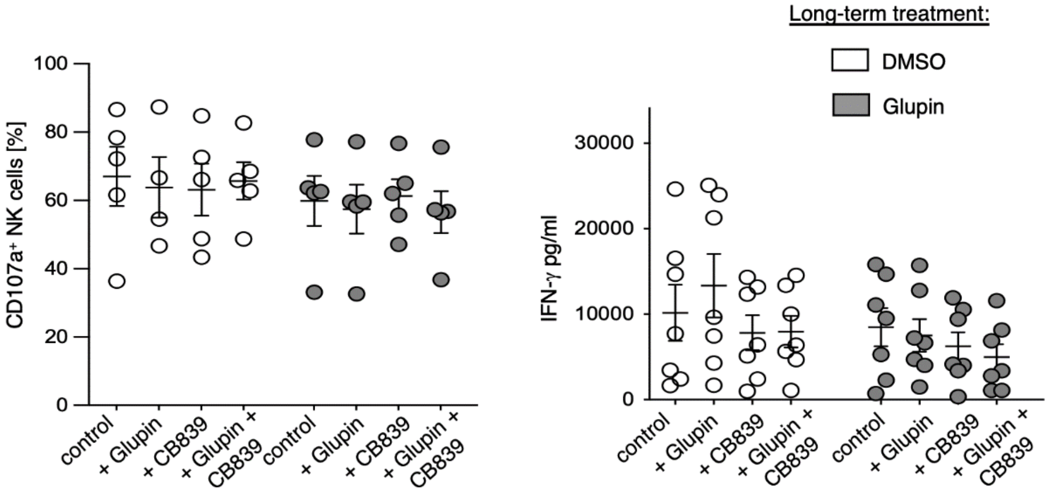

Figure S6. No influence of glutaminase inhibition on the activity of Glupin-treated NK cells. Long-term Glupin or DMSO treated NK cells were washed and treated with 0.1% DMSO, 100 nM Glupin, 0.5 µM CB839 or a combination of 100 nM Glupin + 0.5 µM CB839 for 72 h, followed by stimulation with plate-bound antibodies against CD16. Cells were stimulated for (left) 2 h (CD107a assay) or (right) 16 h (IFN-g). n=5-7 donors from 4 independent experiments. Mean with SEM.
